# Subcortical short‐term plasticity elicited by deep brain stimulation

**DOI:** 10.1002/acn3.51275

**Published:** 2021-04-07

**Authors:** Mohammad Z. Awad, Ryan J. Vaden, Zachary T. Irwin, Christopher L. Gonzalez, Sarah Black, Arie Nakhmani, Byron C. Jaeger, J. Nicole Bentley, Barton L. Guthrie, Harrison C. Walker

**Affiliations:** ^1^ Department of Neurology University of Alabama at Birmingham Birmingham Alabama USA; ^2^ Department of Biomedical Engineering University of Alabama at Birmingham Birmingham Alabama USA; ^3^ Department of Electrical and Computer Engineering University of Alabama at Birmingham Birmingham Alabama USA; ^4^ Department of Biostatistics University of Alabama at Birmingham Birmingham Alabama USA; ^5^ Department of Neurosurgery University of Alabama at Birmingham Birmingham Alabama USA

## Abstract

**Objective:**

To investigate local short‐term neuroplasticity elicited by subthalamic, thalamic, and pallidal deep brain stimulation (DBS) for movement disorders.

**Methods:**

During DBS surgery, we delivered pairs of stimulus pulses with both circular and directional leads across 90 interstimulus intervals in 17 participants and recorded local field potentials from unused contacts on the implanted electrode array. We removed the stimulus artifact, validated the neural origin of the underlying signals, and examined short‐term plasticity as a function of interstimulus interval and DBS target, using linear mixed effects models.

**Results:**

DBS evokes short latency local field potentials that are readily detected with both circular and directional leads at all stimulation targets (0.31 ± 0.10 msec peak latency, mean ± SD). Peak amplitude, area, and latency are modified strongly by interstimulus interval (*P* < 0.001) and display absolute and relative refractory periods (0.56 ± 0.08 and 2.94 ± 1.05 msec, respectively). We also identified later oscillatory activity in the subthalamic‐pallidal circuit (4.50 ± 1.11 msec peak latency) that displays paired pulse facilitation (present in 5/8 subthalamic, 4/5 pallidal, and 0/6 thalamic trajectories, *P* = 0.018, Fisher’s exact test), and correlates with resting beta frequency power (*P* < 0.001), therapeutic DBS frequencies, and stimulation sites chosen later for therapy in the ambulatory setting (*P* = 0.031).

**Interpretation:**

Paired DBS pulses synchronize local circuit electrophysiology and elicit short‐term neuroplasticity in the subthalamic‐pallidal circuit. Collectively, these responses likely represent the earliest detectable interaction between the DBS pulse and local neuronal tissue in humans. Evoked subcortical field potentials could serve as a predictive biomarker to guide the implementation of next‐generation directional and adaptive stimulation devices.

## Introduction

High frequency deep brain stimulation (DBS) improves symptoms of movement disorders when medications are no longer effective. Despite increasing adoption, outcomes vary, and fundamental knowledge about how DBS interacts with human brain circuits is limited. Robust electrophysiology biomarkers could guide clinical innovation with increasingly adaptable and complex device technologies such as closed loop and directional stimulation.

Stimulus pulses in both the subthalamic nucleus (STN) and the ventral intermediate thalamus (VIM) reliably elicit short (~2–3 msec) and long (>5 msec) latency event related potentials (ERPs) measured from the scalp with electroencephalography (EEG) and the cortical surface with electrocorticography (ECoG).^1^, ^2^, ^3^ Stimulus artifact removal reveals faster activation at latencies of ~0.5–2 msec, as well.^2^, ^4^, ^5^ The peak amplitude of these responses correlates with stimulus amplitude and clinical efficacy during high frequency DBS, and the timing and morphology of both short‐ and long‐latency responses can distinguish effective versus ineffective STN stimulation sites within a given patient, both awake and under general anesthesia.^2^, ^4^, ^6^

Prior studies use conventional ring‐shaped leads and often focus on stimulus‐evoked electrophysiology at relatively distant recording sites (scalp, cortical surface) because of ease of access and the smaller electrical transient from the DBS pulse. A few studies have recorded subcortical potentials evoked by DBS that may correlate with clinical efficacy, with responses at latencies between 0.2 and 3 msec in both STN^7^ and VIM^8^ Additionally, bursts of stimulation appear to elicit later resonant responses at ~4 msec latency in the STN, but not in the VIM.^9^, ^10^ These novel subcortical potentials have been proposed as biomarkers to guide DBS therapy, but because of the close temporal proximity and large amplitude of the stimulus artifact, their neural origin remains uncertain.

Here we remove the stimulus artifact and use paired stimuli in multiple brain targets to validate and characterize the earliest local tissue electrophysiology elicited by DBS. Our hypotheses were that (1) robust subcortical responses are present at all targets; (2) these responses display refractory periods and latencies consistent with neuronal activity; (3) pairs of DBS pulses at therapeutically relevant frequencies elicit local short‐term neuroplasticity; and (4) novel directional DBS electrodes provide greater spatial resolution of these phenomena versus conventional circular contacts.

## Materials and Methods

### Participants

Patients were diagnosed with Parkinson’s disease or essential tremor by a movement disorders neurologist based on consensus criteria, and DBS was recommended as part of routine care. This project received prior IRB approval, and all participants signed written consent before enrollment.

### DBS surgery, behavioral assessments, and electrode localizations

We routinely place unilateral DBS contralateral to the most affected side of the body and proceed with staged surgery later (if and when it is needed clinically). These surgeries are conducted under local anesthesia with the patient fully awake. We administer intravenous midazolam 1–2 mg during placement of the stereotactic frame approximately 1.5–2 h prior to the electrophysiological recordings. We target STN and the globus pallidus interna (GPi) with multi‐pass single unit microelectrode recording/stimulation and DBS macrostimulation and target VIM with DBS macrostimulation alone.^11^ Intraoperative O‐arm CT images are co‐registered with brain MRI for targeting and to assess micro‐ and macroelectrode locations, and final DBS electrode location was chosen based on both awake physiology and merged anatomic images. We measured motor symptoms intraoperatively at baseline and during DBS with upper extremity subscores of the Unified Parkinson’s Disease Rating Scale (UPDRS) part 3 upper (“off” medications) and the Fahn‐Tolosa‐Marin Clinical Rating Scale for Tremor (FTM), respectively.^12^, ^13^ In 15 of the 17 patients, we implanted a Medtronic model 3387 lead, and in the remaining 2 we implanted an 8‐segment Abbott model 6173 lead which allowed directional stimulation and sensing. We localized DBS electrodes in midcommissural space on routine postoperative MR images and normalized contact locations based on AC‐PC length, as described previously.^2^, ^4^, ^6^. DBS devices were activated approximately 1 month after surgery as part of routine care by clinicians blinded to the intraoperative electrophysiology. In each participant, we identified active DBS contacts for chronic therapy, defined as stable contact settings across multiple visits at >6 months after surgery.

### Signal acquisition and experimental stimulation

After deactivating non‐essential electrical equipment, we recorded local field potentials with an actiCHamp amplifier (Brain Vision LLC, Morrisville, NC), sampling at 100 kHz with an analog low pass filter of 7.6 kHz, and a ground electrode outside the sterile field on the scalp. Event‐related potentials (ERPs) were elicited at therapeutically effective locations/configurations and stimulus amplitudes, as identified during intraoperative DBS macrostimulation at 160 Hz frequency and 60 *μ*sec pulse width. These clinical effective settings were determined intraoperative during DBS surgery. An external pulse generator (STG4002, MultiChannel Systems, Reutlingen, Germany) delivered monophasic square waves through the DBS lead. Stimuli were delivered through 2 DBS contacts and field potentials nearby unused contacts recorded local field potentials. Anode/cathode contacts and stimulus amplitude were identical to DBS settings that elicited significant clinical efficacy during intraoperative macrostimulation versus preop baseline (Table 2). Pairs of DBS pulses were delivered across 90 unique inter‐stimulus intervals ranging from 0.18 to 30 msec in randomized blocks, with 80 stimulation events in each polarity, yielding exact charge balance across 160 unique stimulation events per block. Pauses between successive pairs of stimuli ranged from 35 to 45 msec in a random, uniform distribution (mean pause = 40 msec). The pulse generator delivered unique TTL signals for each stimulation event to the recording amplifier with sync precision of 10 *μ*sec. We never exceeded the FDA recommended charge density limit of 30 *µ*coul/cm^2^ per phase, and experimental stimulation was behaviorally imperceptible in all participants.

### Digital signal processing

We analyzed the electrophysiology signals with EEGLAB^14^ and custom routines in Matlab (Mathworks, Natick, MA), first re‐referencing the unused circular DBS contacts (typically contacts 2–3) into bipolar configuration to record local signals in close proximity to the stimulation site. In participants with directional leads, we referenced signals from each directional contact segment to the common average.

**Figure 1:**
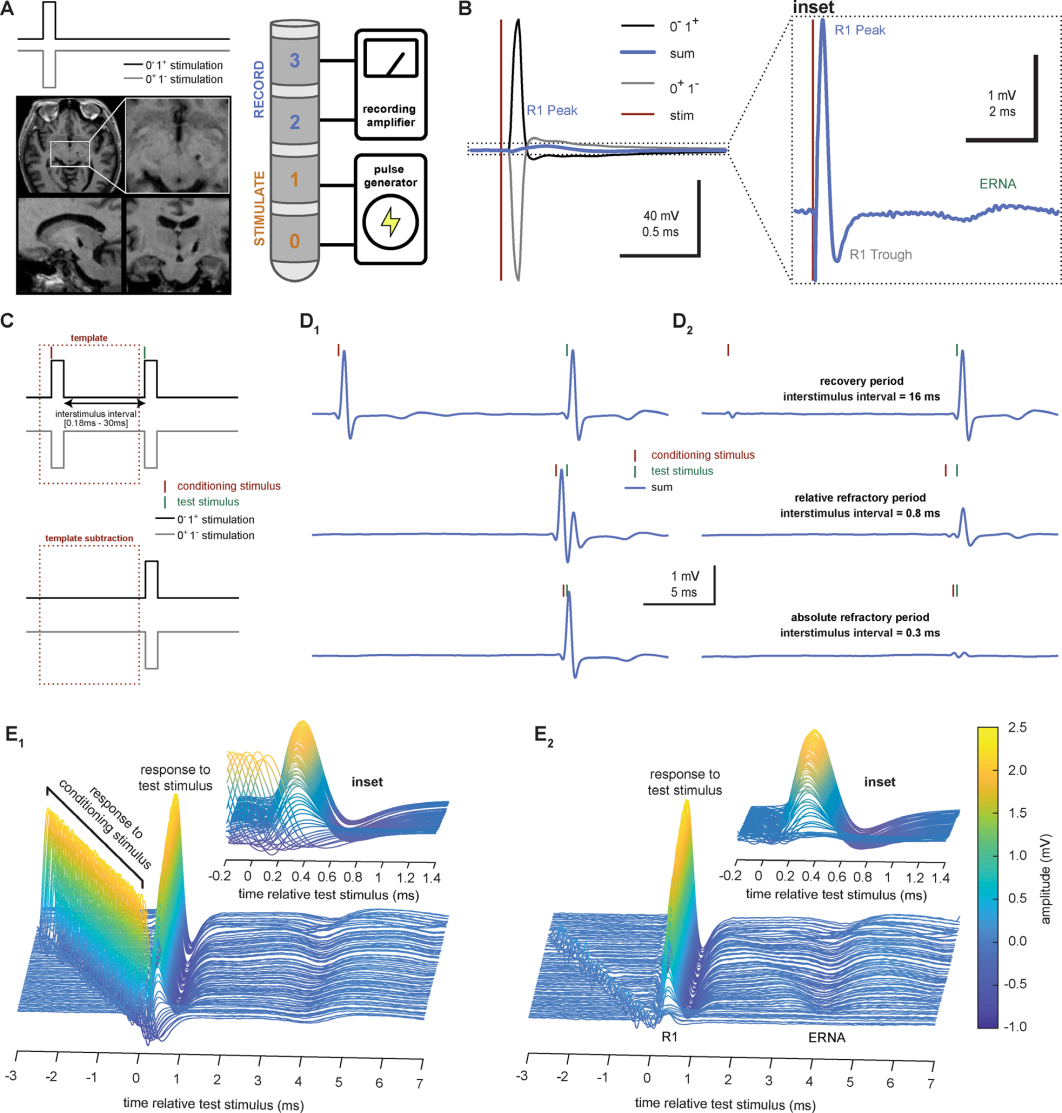
DBS elicits short latency subcortical local field potentials at multiple time scales. (A) Schema showing reversed square wave DBS pulses used for artifact removal, and axial, sagittal, and frontal T1‐weighted MR images showing lead placement in the STN region. (B) Stimuli were delivered from contacts 0 and 1, and local field potentials were recorded from contacts 2 and 3. Reversal of the anode (+) and cathode (−) stimulation contacts inverts stimulus artifact polarity (black and gray traces). Summing these responses eliminates the artifact and reveals an underlying evoked potential (blue trace). Inset shows large amplitude, short latency potentials (R1 peak and trough) evoked by STN DBS, followed by smaller amplitude, more temporally dispersed evoked resonant neural activity (ERNA). (C) Schema for removal of the conditioning stimulus response, which sometimes overlaps the subsequent test stimulus. Template waveform subtraction of the conditioning stimulus isolates the test stimulus response. (D) Subcortical field potentials evoked by pairs of STN DBS pulses, before versus after template subtraction (D1 vs. D2), across three interstimulus intervals. (E) Similar field potentials across 86 unique interstimulus intervals in the same participant. Insets display the test stimulus response before versus after template subtraction (E1 vs. E2).

Two post‐hoc signal processing techniques are required to address both the large stimulus artifact and the remnants of the response to the conditioning stimulus, which overlaps onto the subsequent test stimulus at short interstimulus intervals. First, we remove the stimulus artifact by reversing the anode (+) and cathode (−) contacts on the DBS electrode, which inverts the polarity of the electrical stimulus but not the brain response (Fig. 1A‐B)^1^, ^2^, ^4^, ^6^. We then sum corresponding pairs of stimulus‐evoked potentials (before and after anode/cathode reversal), yielding a de‐artifacted composite evoked potential for each condition. Second, we remove residual activity from the conditioning stimulus by averaging the responses to all conditioning stimuli with interstimulus intervals of ≥20 msec, yielding a template evoked response for each participant (Fig. 1C‐D). We then subtract this template response from the other conditioning stimuli to isolate activity arising from the test stimulus alone, leaving 86 unique paired pulse intervals for subsequent analyses (range 0.18 to 16 msec). We visually inspected the de‐artifacted traces in each participant and only analyzed evoked potentials that displayed absolute and relative refractory periods during paired pulse stimulation.

Our primary interest was to characterize subcortical local field potentials from stimulus onset to <7 msec after the stimulus pulse, including short latency potentials in close temporal proximity to the stimulus and later oscillatory activity (ERNA).^9^, ^10^ We first visualized responses across paired pulse intervals with waterfall and surface contour plots, both within participants and as group means. We then used the Matlab function “findpeaks” to measure peak latency and amplitude over this latency range. To contrast responses at different paired pulse intervals within an individual, we normalized response magnitude to the template waveform of the conditioning stimulus, generating a paired pulse ratio (i.e., test stimulus response/conditioning stimulus response). We defined the onset of the absolute refractory period as the first interstimulus interval among 3 consecutive paired pulse ratios of <0.2 and the relative refractory period as the first interstimulus interval among 3 consecutive paired pulse ratios of >0.98. In the subset of participants with directional DBS electrodes, we rendered local field potentials at rest as continuous wavelet transforms using the Matlab function ‘cwt’ and then correlated resting beta frequency power (14–30 Hz) with the stimulus‐evoked activity across directional DBS contacts.

### Statistical analyses

We performed analyses using R version 3.6.0 or higher (R Foundation for Statistical Computing, Vienna, Austria), specifically leveraging core R functions and packages “tidyverse”,^15^ “image3D”, “corrplot”, “lme4”,^16^ and “multcomp”.^17^ The threshold for significance was 0.05 for all statistical tests.

To account for multiple observations within participants, we used linear mixed effects models and measured changes in dependent variables of interest including integrated area, peak amplitude, peak latency, and paired pulse ratio of the subcortical evoked potentials, with fixed effects such as interstimulus interval, stimulation target, beta spectral power at rest, and paired pulse ratio. For all mixed effects models, random intercepts were included to capture individual heterogeneity in average responses between participants. We conducted nested likelihood ratio tests for fixed effects of interest. If the likelihood ratio test for a fixed effect was statistically significant, we used Tukey’s method for multiple comparisons when to measure pairwise difference tests among levels of the fixed effect.

To test the hypothesis that short‐term subcortical plasticity differs across DBS targets, we contrasted the presence/absence of ERNA at the STN, GPi, and VIM targets with Fisher’s exact test. We also examined whether the cathode contact (−) selected for clinical therapy at ≥6 months after device activation corresponded with the anode‐cathode pair used for experimental stimulation during surgery, as a function of the presence or absence of ERNA. We tested this hypothesis against a two‐tailed binomial probability distribution, assuming chance association of *P* = 0.5. Finally, we visualized correlations between Z‐transformed resting beta power and stimulus‐evoked activity, within and across participants with directional DBS leads.

For additional details on methods, please see Data S1.

## Results

### Demographics and clinical data

Age and duration of disease on the day of surgery were 67.2 ± 11.6 and 10.1 ± 4.8 years, respectively (mean ± SD, *n* = 18), and 7 of the 18 participants were women (39%). Among these, 7 were in the STN for PD, 5 in the GPi for PD and 6 in the VIM for ET, and 11 of 18 (61%) were in the left hemisphere. UPDRS part 3 upper extremity subscores “off” medications improved by 14.0 [10.2–17.9] (mean ± 95% CI, *t* = 8.1, *n* = 12 trajectories) in PD participants, and the Fahn‐Tolosa‐Marin upper extremity subscore changed by 5.3 [2.5 to 8.0] (mean ± 95% CI, *t* = 4.5, *n* = 8 trajectories) in patients with ET (*P* < 0.01, respectively, paired t‐tests). DBS improved contralateral arm function during intraoperative testing with DBS on versus preoperative baseline by 57.2% ± 21.5 at the final electrode location (pooled UPDRS and TMF subscores, *n* = 20 trajectories). Demographic data and chronic DBS settings are provided in Table 1. Experimental DBS settings (amplitude, pulse width, anode/cathode pair) were identified based on clinically effective stimulation parameters during DBS macrostimulation in surgery (Table 2).

**Table 1:** Demographic data and chronic stimulator settings.

Id	dIagnosis	Gender	Age^1^	Disease duration^1^	Target	Hemisphere	Anode	Cathode	Amplitude^1^	Pulse width^1^	Frequency^1^
P01	PD	F	71.7	10.0	STN	Left	3	1	4.2	60	110
P02	PD	F	58.1	10.0	STN	Left	3	1	3.0	90	160
P03	PD	F	35.7	17.0	STN	Right	Case	2	3.2	70	130
P04	PD	M	73.1	12.0	STN	Right	Case	2	3.1	60	160
P05	PD	M	57.4	11.0	STN	Left	2	0	4.8	60	160
P06	PD	M	54.6	3.0	STN	Left	3	2	4.0	60	110
P07^2^	PD	M	75.7	7.2	STN	Left	Case	3B	2.8	60	130
P08	PD	F	72.3	9.9	GPi	Right	Case	3	2.5	60	140
P09	PD	M	72.7	4.9	GPi	Left	1	2/3	3.5	60	160
P10	PD	M	65.3	19.3	GPi	Right	Case	3	3.0	60	160
P11	PD	M	75.0	10.2	GPi	Right	Case	2	2.5	60	130
P12^2^	PD	M	51.7	11.1	GPi	Right	Case	2	2.2	60	130
P13	ET	F	80.6	10.0	VIM	Left	3	1	3.8	60	160
P14	ET	F	76.6	10.0	VIM	Right	3	1	4.5	60	160
P15	ET	F	71.4	4.0	VIM	Left	1	3	4.0	60	160
P16	ET	M	80.6	20.0	VIM	Left	3	1	3.2	60	140
P17	ET	M	71.5	4.0	VIM	Left	Case	1	2.7	60	140
P18	ET	M	66.0	9.0	VIM	Left	3	2	4.0	60	130
Mean			67.2	10.1					3.4	62	143
SD			11.6	4.8					0.7	7	18

^1^ Age and duration in years, amplitude in Volts, pulse width in microseconds, frequency in Hertz.

^2^ Abbott directional lead model 6173 with ring, directional, directional, ring configuration (1‐3‐3‐1), numbered 1 to 4, rather than 0 to 3, by convention. Stimulus amplitudes converted from current to voltage.

**Table 2:** Clinical behavioral physiology during DBS surgery.

ID	Target	Recording trajectory	Recording channel(s)	Stimulation channel	Experimental current (mA)	Pre^1^	Macro‐stim^1^	Percent change^1^	Paired pulse stimulation	Directional lead
P01	STN	1	2‐3	0‐1	1.6	34.5	14.5	58.0	Yes	No
P02	STN	1	0‐1	2‐3	1.5	NA	NA	NA	Yes	No
P02	STN	2	0‐3	1‐2	2.1	23.0	10.0	56.5	Yes	No
P03	STN	1	2‐3	0‐1	2.1	22.0	10.0	54.5	Yes	No
P04	STN	1	2‐3	0‐1	1.9	15.0	7.5	50.0	Yes	No
P05	STN	1	2‐3	0‐1	1.3	30.0	12.0	60.0	Yes	No
P06	STN	1	0‐3	1‐2	1.2	18.0	12.0	33.3	Yes	No
P07^2^	STN	1	2A,2B,2C, 3A,3B,3C	1‐4	2.5	8.0	2.0	75.0	Yes	Yes
P08	GPi	1	0‐2	1‐3	NA	26.5	11.5	56.6	No	No
P09	GPi	1	1‐2	0‐3	2.5	26.0	4.0	84.6	No	No
P10	GPi	1	1‐2	0‐3	2.7	20.0	9.5	52.5	No	No
P11	GPi	1	1‐2	0‐3	1.9	29.0	5.5	81.0	No	No
P12^2^	GPi	1	2A,2B,2C, 3A,3B,3C	1‐4	3.0	21.0	6.0	71.4	Yes	Yes
P13	VIM	1	2‐3	0‐1	2.2	9.5	3.0	68.4	Yes	No
P14	VIM	1	2‐3	0‐1	2.7	6.0	4.0	33.3	Yes	No
P14	VIM	2	2‐3	0‐1	3.0	6.0	3.0	50.0	Yes	No
P15	VIM	1	2‐3	0‐1	1.5	12.0	2.0	83.3	Yes	No
P16	VIM	1	2‐3	0‐1	2.1	11.0	0.5	95.5	Yes	No
P17	VIM	1	2‐3	0‐1	2.0	7.5	4.0	46.7	Yes	No
P18	VIM	1	2‐3	0‐1	2.6	21.0	17.5	16.7	Yes	No
P18	VIM	2	2‐3	0‐1	2.6	21.0	17.5	16.7	Yes	No
mean					2.1	18.4	7.8	57.2		
sd					0.5	8.6	5.2	21.5		

^1^ Subscores of the Unified Parkinson's Disease Rating Scale (UPDRS) for PD patients and the Fahn‐Tolosa‐Marin Clinical Rating Scale for Tremor, during macrostimulation versus prior to implant.

^2^ Abbott directional lead model 6173 with ring, directional, directional, ring configuration (1‐3‐3‐1), numbered 1 to 4, rather than 0 to 3, by convention.

### DBS‐evoked subcortical electrophysiology: response dynamics and short‐term plasticity

Artifact removal can be challenging because of the proximity of the stimulation and recording sites. We therefore only analyze data from 19 of 21 trajectories (90%) whose evoked potentials showed clear absolute and relative refractory periods in response to paired stimulus pulses or a clear response to the single pulse stimulation with an amplitude above the noise level.

**Figure 2:**
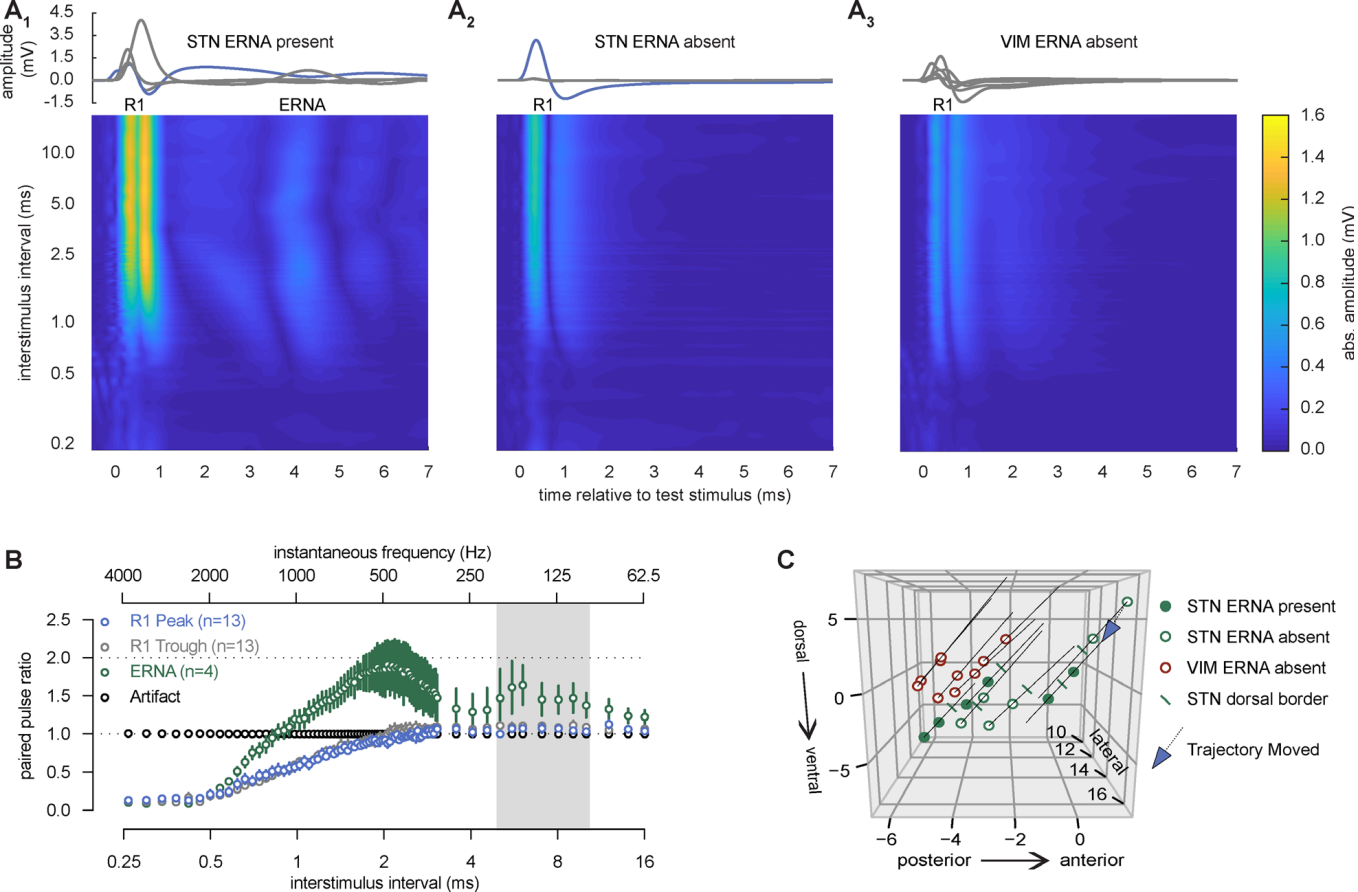
STN DBS elicits short‐latency neuroplasticity in adjacent subcortical tissue. (A) Traces of stimulus‐evoked subcortical potentials (within each participant) and group‐level fully rectified mean contour plots demonstrate that a subset of STN (A1) but not VIM (A3) stimulation sites elicited ERNA. Note that ERNA was absent initially in one participant, and later emerged only after moving the DBS lead to its final location during surgery (blue trace in A2 versus A1). Associated group level contour plots display relative and absolute refractory periods at short interstimulus intervals (~2.5 to 0.6 msec and <0.6 msec, respectively) (B) In contrast to the stimulus artifact, R1 and ERNA area are modulated by the timing of the prior DBS pulse (mean ± SEM). R1 and ERNA both display absolute and relative refractory periods, but only ERNA shows short‐term facilitation at specific interstimulus intervals (*χ* ^2^ (1) = 318.2, *P* < 0.001). The gray boxed background represents the range for clinically effective frequency for DBS (100–200 Hz). (C) Stimulation sites with versus without ERNA, in midcommissural space, superimposed on DBS lead reconstructions (STN and VIM implants only). Blue arrow indicates the participant from panel A who only displayed ERNA upon moving the lead to a more effective stimulation site during surgery. DBS lead localization was not measured in three participants because post‐operative MR images were unavailable.

DBS pulses at all targets elicit short latency, large amplitude local field potentials (denoted R1) with peak and trough latencies and amplitudes of 0.31 ± 0.10 msec and 1.10 ± 0.93 mV, and 0.72 ± 0.11 msec and 0.54 ± 0.44 mV, respectively (*n* = 19) (Table 3, Figs. 1 and 2). R1 peak amplitude did not differ by stimulation target (STN, GPi, VIM) at our level of statistical power (mixed effects linear model with dependent variable R1 peak amplitude and fixed effects interstimulus interval and DBS target, *χ* ^2^ (1) = 3.6, *P* = 0.056, Fig. 2).

**Table 3:** Subcortical electrophysiology evoked by DBS.

ID	Target	Traj	R1 peak amplitude^1^	R1 peak latency^1^	R1 trough amplitude^1^	R1 trough latency^1^	ARP^1^	RRP^1^	ERNA amplitude^2^	ERNA latency^2^, ^3^
P01	STN	1	2.19	0.28	0.61	0.67	0.56	2.36	0.16	4.30
P02	STN	2	1.04	0.33	0.86	0.75	0.64	2.76	0.20	4.39
P05	STN	1	3.58	0.58	Absent	Absent	0.60	2.44	0.72	4.34
P06	STN	1	1.14	0.29	0.26	0.72	0.64	2.56	0.17	3.76
P07^2^	STN	1	1.90	0.27	Absent	Absent	0.50	2.88	0.24	4.59
P08	GPi	1	1.62	0.25	1.66	0.66	NA	NA	0.34	7.29
P10	GPi	1	0.86	0.20	0.39	0.58	NA	NA	0.15	3.39
P11	GPi	1	0.74	0.31	Absent	Absent	NA	NA	0.12	4.48
P12^2^	GPi	1	0.97	0.36	0.40	0.65	0.47	1.70	0.10	3.96
P02	STN	1	2.49	0.35	1.00	1.00	0.68	3.62	Absent	Absent
P03	STN	1	0.07	0.28	0.03	0.67	0.40	2.36	Absent	Absent
P04	STN	1	0.12	0.31	0.03	0.72	0.48	2.36	Absent	Absent
P09	GPi	1	0.64	0.20	0.43	0.61	NA	NA	Absent	Absent
P13	VIM	1	1.69	0.35	1.21	0.81	0.56	2.40	Absent	Absent
P14	VIM	1	0.30	0.20	0.36	0.65	0.60	4.12	Absent	Absent
P14	VIM	2	0.26	0.17	0.35	0.62	0.68	6.12	Absent	Absent
P16	VIM	1	0.16	0.31	0.08	0.74	0.54	2.52	Absent	Absent
P17	VIM	1	0.73	0.33	0.57	0.73	0.60	2.76	Absent	Absent
P18	VIM	1	0.47	0.48	0.47	0.86	0.48	3.12	Absent	Absent
mean			1.10	0.31	0.54	0.72	0.56	2.94	0.24	4.50
sd			0.93	0.10	0.44	0.11	0.08	1.05	0.19	1.11

Abbreviations: recording trajectory (traj), absolute refractory period (ARP), relative refractory period (RRP), and evoked resonant neural activity (ERNA).

^1^ Amplitude in mV; latencies and refractory periods in ms.

^2^ Responses are mean across directional DBS contacts within each participant.

^3^ Largest peak between 3 and 7 ms post‐stimulus latency.

STN and GPi DBS, but not VIM DBS, elicit resonant oscillatory potentials at longer latencies, as well. These later potentials (termed evoked resonant neuronal activity or ERNA) occurred at peak latencies and amplitudes of 4.50 ± 1.11 msec and 0.24 ± 0.19 mV, respectively, in 5/8 STN (63%), 4/5 GPi (80%), and 0/6 VIM (0%) trajectories (*P* = 0.018, Fisher’s exact test) (Figs. 2A and 5F). In contrast to R1, paired pulse stimulation demonstrates short‐term facilitation of ERNA, but not R1, across two ranges of interstimulus intervals (~1–4 msec and ~5–10 msec) (dependent variable paired pulse ratio and fixed effects peak/trough/ERNA and interstimulus interval, *χ* ^2^ (1) = 318.2, *P* < 0.001) (Fig. 2B). STN trajectories with and without ERNA clustered more anteriorly and ventrally in midcommissural space versus VIM trajectories (Fig. 2C).

**Figure 3:**
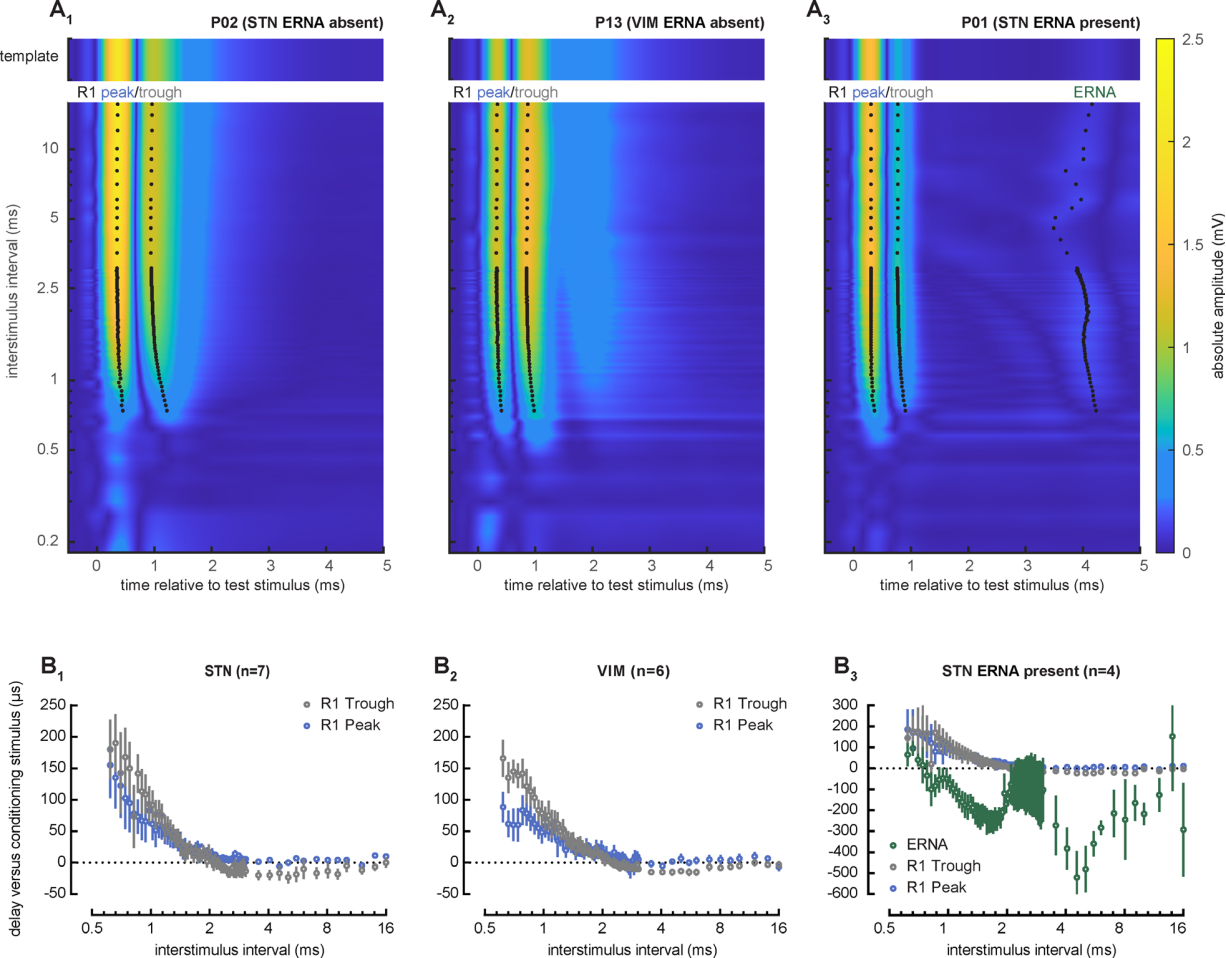
Prior stimulus history alters the timing of short latency subcortical field potentials elicited by DBS. (A1–A3) Fully rectified contour plots of subcortical stimulus‐evoked activity as a function of interstimulus interval, from representative participants in the VIM and STN targets. As interstimulus interval shortens, R1 peak and trough latency increase, regardless of DBS target. In contrast, ERNA latency at its maximum amplitude is more variable and becomes shorter at specific paired pulse intervals. R1 and ERNA both progressively diminish beginning at interstimulus intervals <2.5 msec and become undetectable/absent at <0.6 msec. (B1–B3) R1 trough becomes slightly more delayed than R1 peak within the relative refractory period, regardless of DBS target, with a significant interaction with interstimulus interval (*χ* ^2^ (1) = 4.7, *P* = 0.031 and *χ* ^2^ (1) = 15.0, *P* < 0.001, respectively). In STN participants with ERNA, R1 peak and trough latencies become delayed as well, whereas ERNA latency shortens and displays greater variability as a function of interstimulus interval (*χ* ^2^ (1) = 797.4, *P* < 0.001).

Response latency is modulated differentially by prior stimulation history. R1 latency become progressively delayed as the paired pulse interval enters the relative refractory period (Fig. 3), with slightly greater delays in R1 trough versus peak latency as a function of interstimulus interval (dependent variable change in latency versus conditioning response, with fixed effects peak/trough and peak/trough : interstimulus interval interaction, *χ* ^2^ (1) = 4.7, *P* = 0.031 and *χ* ^2^ (1) = 15.0, *P* < 0.001, respectively) (Fig. 3B1 and B2). In contrast to R1, ERNA latencies shorten across a range of interstimulus intervals (dependent variable change in latency, and fixed effects peak/trough/ERNA and interstimulus interval, *χ* ^2^ (1) = 797.4, *P* < 0.001) (Fig. 3B3). Further, hastening of ERNA correlates significantly with its degree of paired pulse facilitation, and not with interstimulus interval (dependent variable change in ERNA peak latency, with fixed effects ERNA paired pulse ratio and interstimulus interval, *χ* ^2^ (1) = 42.4, *P* < 0.001 and *χ* ^2^ (1) = 1.8, *P* = 0.18, respectively).

### Resonant oscillatory activity in the STN‐GPi circuit: sensing directional local field potentials

**Figure 4:**
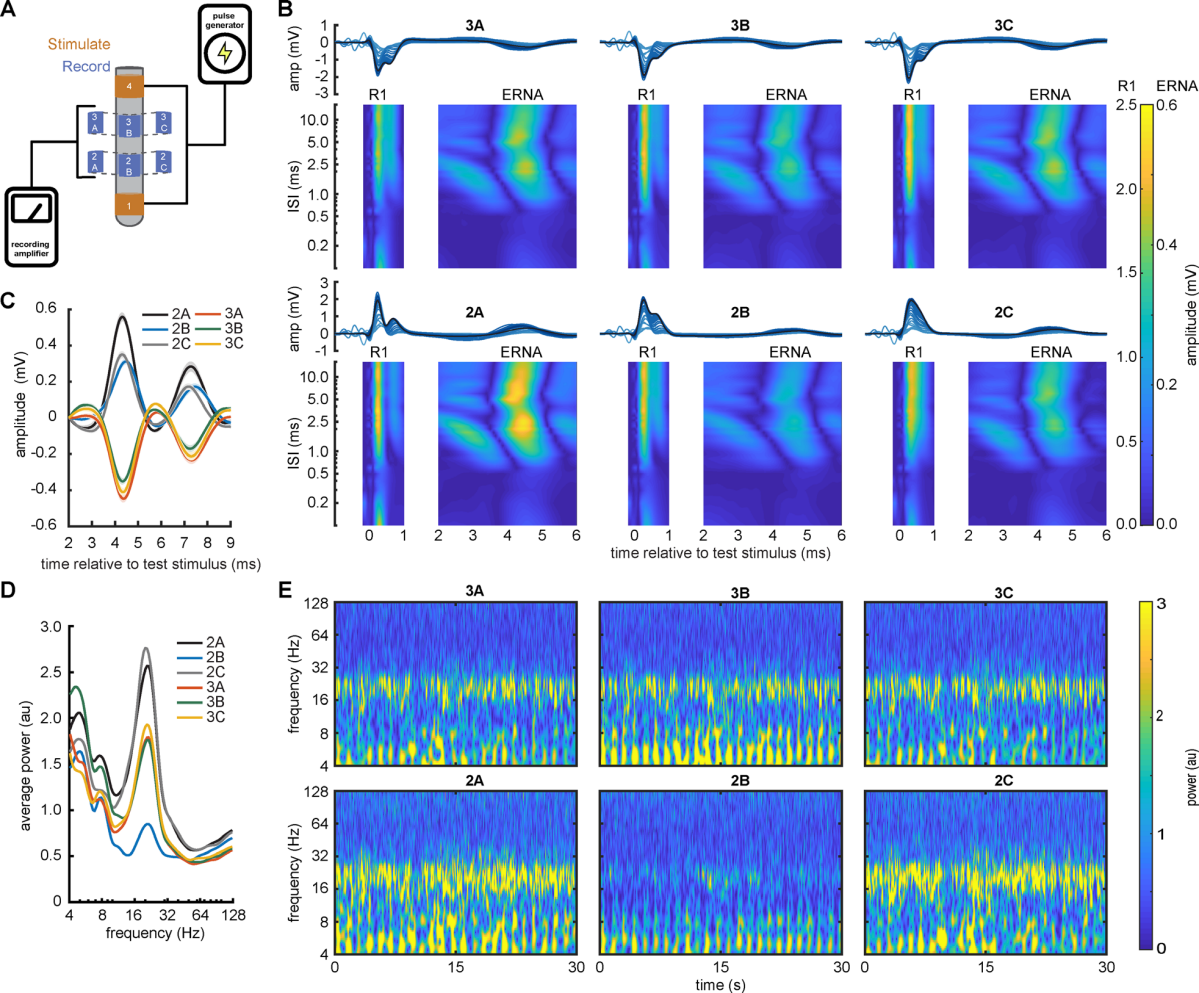
Stimulus‐evoked and spontaneous STN oscillations display spatial gradients across directional DBS electrode contacts. (A) Stimulation and recording configuration for the directional DBS lead. (B) Directional subcortical evoked potentials elicited by pairs of STN DBS pulses with corresponding fully rectified contour plots. Traces display phase reversals between the upper and lower row of DBS contacts, and contour plots demonstrate spatial gradients across DBS contact segments. (C) ERNA displays phase reversals across DBS contact rows (mean ± SEM). (D, E) Resting beta power varies across directional DBS contacts (mean and continuous wavelet spectrograms).

**Figure 5:**
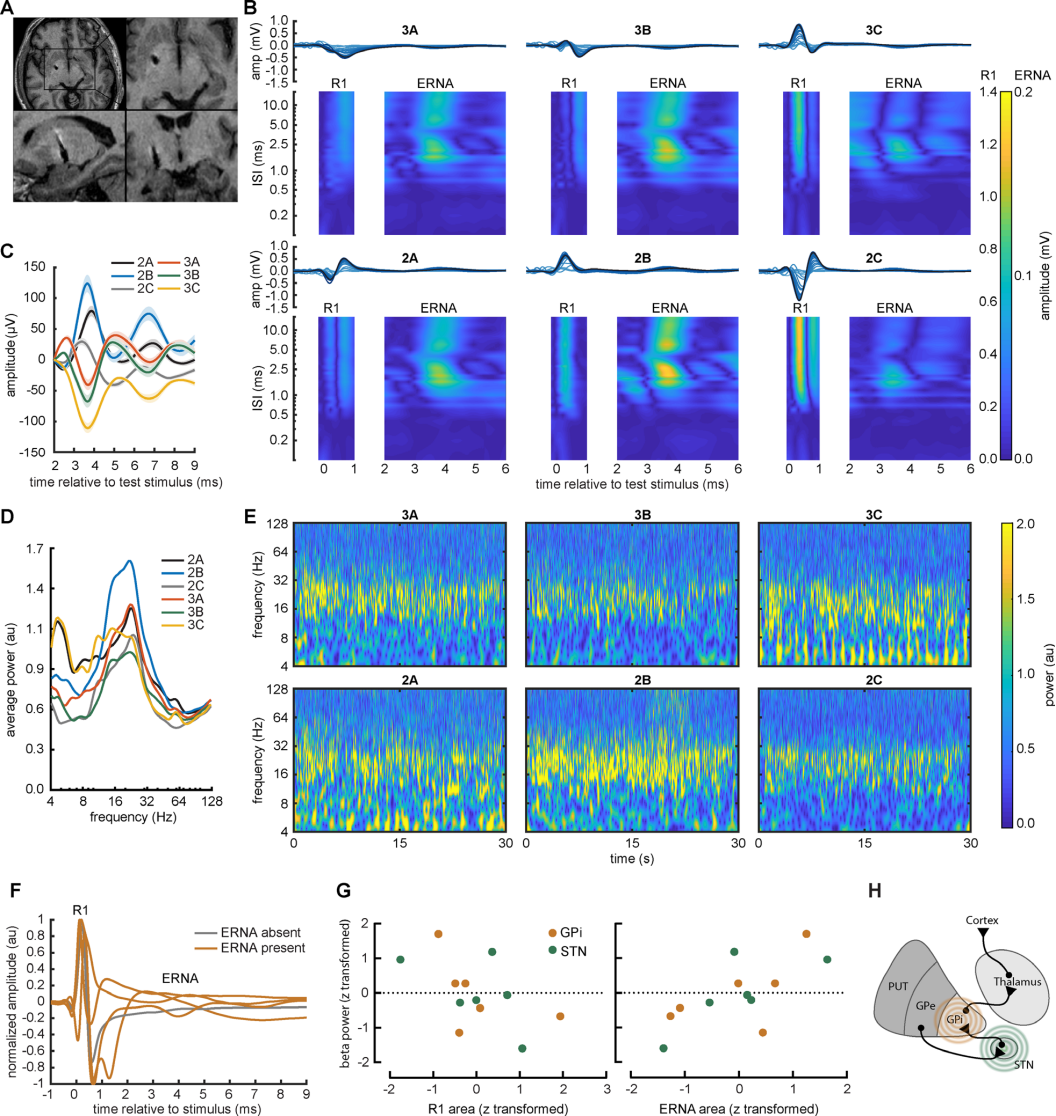
Stimulus‐evoked and spontaneous oscillations in the STN/GPi circuit. (A) Axial, sagittal, and frontal T1‐weighted MR images show lead placement in the GPi region. (B) Pairs of GPi stimuli evoke directional local field potentials. Traces display phase reversals between specific directional contact segments, and traces and rectified contour plots both demonstrate spatial gradients across the directional lead. (C) Magnified view of ERNA phase reversals across directional DBS contacts (mean ± SEM). (D, E) Resting beta power varies across directional contacts (mean and continuous wavelet spectrograms). (F) GPi DBS elicited ERNA in 4/5 participants (80%). (G) Resting beta power correlates negatively with both R1 area and positively with ERNA area from the template waveform (all values are z‐normalized within each participant for visualization) (mixed effects linear model *χ* ^2^ (1) = 6.7 and *P* = 0.010; and *χ* ^2^ (1) = 25.5 and *P* < 0.001, respectively). (H) Abbreviated network diagram for ERNA in the STN → GPI circuit.

We examined spontaneous and evoked field potentials in two participants with directional DBS leads at rest and in response to STN and GPi stimulus pulses, again using clinically effective stimulation parameters identified during DBS surgery (Figs. 4 and 5). Stimulation was delivered from the outer ring contacts and recordings were obtained from the unused directional contacts (Fig. 4A). R1 and ERNA were present across all directional contacts in both STN and GPi. R1, ERNA, and resting beta power all displayed spatial gradients across directional DBS contact segments within a given participant. R1 and ERNA amplitudes and resting beta power vary across directional DBS contacts within individuals (Figs. 4C and D and 5C and D). ERNA was present at both stimulation targets and displayed similar refractory periods and short‐term plasticity phenomena versus ring‐shaped leads. In our sample, resting beta power correlates positively with ERNA area, and negatively with both R1 area (dependent variable resting beta power and fixed effects ERNA area and R1 area *χ* ^2^ (1) = 25.5, *P* < 0.001; and *χ* ^2^ (1) = 6.7, *P* = 0.010, respectively, Fig. 5G). In the GPi target, ERNA was present in 4/5 (80%) subjects (Fig. 5F).

### DBS‐evoked short‐term plasticity: clinical translation

We examined the potential clinical relevance of these findings by associating short‐term plasticity with the location and the timing of the paired stimuli. Clinicians blinded to the intraoperative electrophysiology selected STN DBS contacts for chronic therapy that elicited ERNA during surgery, whereas STN DBS contacts without ERNA were not selected for therapy (6/6 concordance, *P* = 0.039, two‐tailed binomial probability distribution, Table 4). Anatomic boundaries within each recording trajectory (Table S1) were used to assign an anatomical region to each DBS contact in Table 4. Similar analyses were not conducted on directional and GPi participants because we utilized a broad stimulation field that spanned the entire range of the DBS electrode contacts. Finally, we observed greater paired pulse facilitation at interstimulus intervals that correspond with clinically effective DBS frequencies (5–10/100–200 Hz) versus longer intervals at ineffective frequencies (12–16 msec/~60–80 Hz) (Fig. 2B).

**Table 4:** DBS contacts anatomical designations based on subcortical single unit recordings, evoked LFPs, and DBS contacts chosen for DBS therapy.

ID	Target	Traj	DBS0	DBS1	DBS2	DBS3	Stim channels (OR)	ERNA present (OR)	Final cathode (clinic)
P01	STN	1	STN	STN	ZI	thal	0‐1	Yes	1
P02^1^	STN	1	STN	STN	ZI	thal	2‐3	No	NA
P02^1^	STN	2					1‐2	Yes	1
P03	STN	1	STN	STN	ZI	thal	0‐1	No	2
P04	STN	1	STN	ZI	ZI	thal	0‐1	No	2
P05	STN	1	STN	STN	STN	ZI/thal	0‐1	Yes	0
P06	STN	1	STN	STN	ZI	thal	1‐2	Yes	2
P07^2^	STN	1	STN	STN	STN	ZI/thal	1‐4	Yes	3b
P08	GPi	1	GPi	GPi	GPi/GPe	GPe	1‐3	Yes	3
P09	GPi	1	GPi	GPi/GPe	GPe	GPe	0‐3	No	2/3
P10	GPi	1	GPi	GPi	GPe	GPe	0‐3	Yes	3
P011	GPi	1	GPi	GPi	GPi	GPi	0‐3	Yes	2
P12^2^	GPi	1	GPi	GPi	GPi/GPe	GPe	1‐4	Yes	2
							6/6 concordance of adjacent bipolar pairs (shaded gray) p=0.031, two‐tailed binomial test^2^		

Abbreviations: recording trajectory (traj), thalamus (thal), zona incerta (ZI), subthalamic nucleus (STN), substantia nigra pars reticulata (SNR), anterior thalamus (thal), globus pallidus externus (GPe), globus pallidus internus (GPi), evoked resonant neural activity (ERNA).

^1^ Microelectrode recordings were not obtained for the second trajectory.

^2^ Participants with directional leads were not included in the binomial test because we delivered a broader bipolar stimulation field from outer rows (contacts 1 and 4) that surrounded the directional contact segments in the middle rows (2a, 2b, 2c, 3a, 3b, 3c) of the DBS lead.

## Discussion

Here we characterize subcortical short‐term neuroplasticity evoked by DBS in patients with movement disorders. DBS elicits complex local circuit dynamics at multiple time delays, including short latency potentials that likely represent the earliest detectable electrophysiological response to DBS in humans when recording from extracellular macroelectrodes. These findings have significant therapeutic and mechanistic implications, suggesting that paired DBS pulses synchronize local tissue electrophysiology and evokes short‐term facilitation in the STN‐GPi circuit. Furthermore, they validate, synthesize, and expand on prior work in this domain from other groups.^8^, ^9^, ^10^ Subcortical potentials elicited by DBS could eventually serve as a biomarker to estimate dose or circuit engagement, with potential to guide therapy either during surgery or with next‐generation directional or adaptive DBS devices.

Based upon its short latency, fixed timing, and refractory periods, R1 most likely represents non‐synaptic, direct activation of local neuronal elements from the stimulus pulse. Accurate removal of the stimulus artifact and paired stimulus pulses are key technical hurdles that allow robust visualization and validation of these signals, including direct measurement of absolute and refractory periods in 19/21 trajectories (90%). These physiological parameters are of considerable interest, particularly in the context of DBS therapy in humans. Our estimates of the refractory periods agree with prior work in humans and a variety of animal models.^18^, ^19^ Additionally, others have found similar delays in response latency during the relative refractory period.^20^ Presumably, R1 latency becomes delayed because of relative inactivation of voltage‐gated sodium channels, such that an identical stimulus takes longer to sufficiently charge the extracellular membrane to elicit an action potential.

In contrast, later oscillatory response (ERNA) most likely represents an orthodromic, synaptic activity. This inference is supported by several observations. First, the longer latency of ERNA (typically >3 msec, mean 4.50 ± 1.11 msec) is compatible with time delays for signal conduction and synaptic activity. Second, in contrast to R1, ERNA displays paired pulse facilitation, a form of short‐term plasticity, across two broad ranges of interstimulus intervals (~2–4 msec and 5–10 msec). Third, ERNA amplitude and latency are more variable than the earlier responses, consistent with the known temporal dynamics of synaptic vesicle release. In particular, the hastening of ERNA at specific interstimulus intervals (Fig. 3B3) suggests the precise timing of the prior stimulation history may prime the local circuit to more easily recruit a subsequent response. Speculatively, short‐term neuroplasticity in the STN/GPi circuit could represent interactions with projections from cortex or GPe, intrinsic properties of the STN or GPi neurons themselves (Fig. 5H), or other mechanisms.^9^, ^10^, ^21^

Our findings have implications for DBS mechanism of action. Contrary to predictions from stereotactic lesions (i.e., thalamotomy, subthalamotomy), functional imaging studies show increased activity in STN and thalamus and reduced activity in frontal cortex during DBS versus no stimulation.^22^, ^23^ Similarly, studies in the primate GPi show single unit activation, not inhibition, during STN stimulation,^24^ and our prior EEG studies show short latency stimulus‐evoked activation at longer delays (~1 msec peak latency) during clinically effective STN and VIM stimulation, presumably from retrograde (antidromic) activation of cortical axons with projections into the stimulation site.^2^, ^4^ Our current findings are broadly consistent with these results, showing earlier depolarization of subcortical elements near the active DBS contacts (0.31 ± 0.10 msec peak latency). Furthermore, the duration of the relative refractory period (<4 msec) suggests that clinically effective DBS frequencies (100–200 Hz) may activate and synchronize local brain circuit electrophysiology to the DBS frequency during chronic therapy.

Our findings have several clinical implications, as well. First, short‐term plasticity was associated with stimulation sites that were later selected for therapy (Table 4). Second, substantial ERNA facilitation occurred at interstimulus intervals (~5–10 msec) corresponding with the timing of effective therapeutic stimulation frequencies (100–200 Hz) (Fig. 2B). Third, R1, ERNA, and the beta frequency band all displayed spatial gradients across directional DBS contact segments within participants, suggesting potential roles for surgical targeting or contact selection during postoperative programming (Figs. 4 and 5). Fourth, the large amplitude of the subcortical evoked potentials (R1, ERNA) versus other modalities (spontaneous EEG and cortical/subcortical LFPs), suggests potential viability as a guide for intraoperative targeting or as control signals for closed‐loop and directional DBS devices.^2^, ^4^, ^25^, ^26^, ^27^, ^28^ Speculatively, the presence of R1 at all stimulation sites implies a potential role in tremor cessation (a shared behavioral response across targets), while short‐term plasticity at longer latencies in ERNA in the STN/GPi circuit might be associated with improvement in rigidity, bradykinesia, and dyskinesias at those targets.

Our study has several strengths and some potential limitations. First, the surgical arena accommodates externalized hardware with enough bandwidth and sync precision to perform these experiments. Future studies should evaluate fully implanted DBS devices with these technical capabilities. Second, surgery can be associated with microlesion effects from acute lead placement that could alter either the behavioral or electrophysiological response to DBS. To address this, we verified that the stimulus location (Table 4), contact configuration, amplitude, and pulse width for the experiments were both clinically effective and well‐tolerated during high frequency DBS (Table 2). Third, although our findings provide evidence for resonant short‐term plasticity in the STN/GPi circuit, these structures receive mixed excitatory and inhibitory inputs from cortex, thalamus, GPe, and other structures. Therefore, without single unit recordings or pharmacologic/optogenetic manipulations, we cannot make direct inferences regarding the specific anatomic basis of these interactions or whether the net effect on the local circuit is excitation, inhibition, or a combination of both. Fourth, we did not evaluate paired pulse intervals of >16 msec or more prolonged stimulus trains, so our findings do not directly address plasticity over these longer time scales. Fifth, although the paired pulse paradigm provides strong internal validation within participants, group level inferences should be interpreted with caution because of limited sample size, although our results contrasting the STN versus VIM target agree with work from an independent group.^9^, ^10^ Sixth, we primarily evaluated stimulation sites and parameters that were clinically effective during surgery; future studies should directly contrast effective versus ineffective stimulation parameters and locations. Seventh, our peak amplitude estimates are derived from the sum of two evoked potentials (i.e., 1+ 0− plus 1‐0+), thus they likely overestimate the response amplitude to either condition alone. Finally, in most cases we stimulated and recorded from the ventral and dorsal pair of DBS contacts, respectively, and although we sensed the evoked LFPs with directional leads we delivered stimulation exclusively with ring‐shaped electrodes. Modification of the stimulation/recording configuration and the use of DBS leads with different geometries might yield different results.

## Conclusions

Pairs of DBS pulses at therapeutic frequencies synchronize local tissue electrophysiology at multiple time scales and elicit short term neuroplasticity in the subthalamic‐pallidal circuit that correlates with both clinical efficacy and beta frequency power. Collectively, these potentials likely represent the earliest detectable electrophysiological interactions between the DBS pulse and local neuronal tissue in humans. Subcortical stimulus‐evoked neuroplasticity has broad potential to guide clinical innovation with next‐generation directional and adaptive DBS technologies.

## Conflict of Interest

Dr. Walker receives research funding from the National Institutes of Health (UH3 NS100553), the Michael J. Fox Foundation, and funding for fellowship training from Medtronic. Dr. Walker also serves as a consultant for Medtronic and Boston Scientific.

## Supporting information


**Table S1.** Subcortical anatomic boundaries based on intraoperative single unit recordings.

**Data S1.** Supplemental method.
