# Supplementary material for: Subcortical short‐term plasticity elicited by deep brain stimulation
Source: Ann Clin Transl Neurol. 2021 Apr 7;8(5):1010–23. doi: 10.1002/acn3.51275 (PMC8108424; doi:10.1002/acn3.51275)
Supplement: Supplementary file 1 — Table S1. Subcortical anatomic boundaries based on intraoperative single unit recordings. [file ACN3-8-1010-s001.docx]

| **Table S1: Subcortical anatomic boundaries based on intraoperative single unit recordings** | | | | | | | | | | | | |
| --- | --- | --- | --- | --- | --- | --- | --- | --- | --- | --- | --- | --- |
| **ID** | **target** | **traj** | **dorsal thal*** | **ventral thal*** | **dorsal ZI*** | **dorsal STN*** | **dorsal SNR*** | **dorsal striat*** | **ventral striat*** | **dorsal GPe*** | **dorsal GPi*** | **ventral GPi*** |
| P01 | STN | 1 | 20.0 | 10.0 | 8.0 | 6.0 | 0.0 |  |  |  |  |  |
| P02** | STN | 1 | 16.0 | 8.0 |  | 7.5 | 1.0 |  |  |  |  |  |
| P02** | STN | 2 |  |  |  |  |  |  |  |  |  |  |
| P03 | STN | 1 | 20.0 | 8.0 | 7.0 | 6.0 | 0.0 |  |  |  |  |  |
| P04 | STN | 1 | 16.0 | 7.0 | 5.5 | 3.0 | -1.0 |  |  |  |  |  |
| P05 | STN | 1 |  |  |  | 7.0 | -1.0 |  |  |  |  |  |
| P06 | STN | 1 | 20.0 | 10.0 | 8.0 | 6.0 | 0.2 |  |  |  |  |  |
| P07 | STN | 1 | 16.0 |  |  | 7.0 | 1.4 |  |  |  |  |  |
| P08 | GPi | 1 |  |  |  |  |  | 20.0 | 12.0 | 10.0 | 6.5 | 0.0 |
| P09 | GPi | 1 |  |  |  |  |  | 16.0 | 15.0 | 14.0 | 7.0 | 3.0 |
| P10 | GPi | 1 |  |  |  |  |  | 20.0 | 12.0 | 10.0 | 4.5 | -1.0 |
| P011 | GPi | 1 |  |  |  |  |  | 19.0 | 14.0 | 12.0 | 9.6 | 2.0 |
| P12 | GPi | 1 |  |  |  |  |  | 16.0 | 10.0 | 10.0 | 6.0 | -1.0 |
| mean |  |  | 18.0 | 8.6 | 7.1 | 6.1 | 0.1 | 18.2 | 12.6 | 11.2 | 6.7 | 0.6 |
| sd |  |  | 2.2 | 1.3 | 1.2 | 1.5 | 0.9 | 2.0 | 1.9 | 1.8 | 1.9 | 1.8 |
| * distance from target in mm. ** microelectrode recordings were not obtained for the second trajectory. Abbreviations: recording trajectory (traj), thalamus (thal), zona incerta (ZI), subthalamic nucleus (STN), substantia nigra pars reticulata (SNR), anterior thalamus (thal), striatum (striat), globus pallidus externus (GPe), globus pallidus internus (GPi). | | | | | | | | | | | | |
