# Supplementary material for: Subcortical short‐term plasticity elicited by deep brain stimulation
Source: Ann Clin Transl Neurol. 2021 Apr 7;8(5):1010–23. doi: 10.1002/acn3.51275 (PMC8108424; doi:10.1002/acn3.51275)
Supplement: Supplementary file 2 — Data S1. Supplemental method. [file ACN3-8-1010-s002.docx]

**Supplemental Materials**

**Supplemental Methods**

*Signal acquisition and electrode used for recording.*

In 15 of the 17 patients we implanted a Medtronic model 3387 lead (a linear array of four ring-shaped contacts numbered from 0 to 3 with width of 1.5 mm and separated by 1.5 mm), whereas we implanted an 8-segment Abbott model 6173 directional lead in 2 patients (a four-row lead in a 1-3-3-1 configuration containing 3 equally spaced DBS contacts segments in the middle two rows, each row separated by 1.5 mm; Abbott, Abbott Park, IL.). For circular DBS leads, we typically delivered bipolar stimuli through the ventral contacts (0-1) and recorded evoked potentials from the unused dorsal contacts (2-3), save for six participants in whom we stimulated through contacts 1-2, 2-3, 1-3, or 0-3 and recorded from contacts 0-3, 0-1, 0-2, or 1-2 respectively, based on the results of clinical macrostimulation. In two participants with directional DBS leads, we delivered bipolar stimuli from the outer ring contacts and recorded field potentials from the directional contact segments. In 3 of 17 participants, we delivered low frequency stimulation of 20 Hz without paired pulses and examined the presence or absence of the earliest evoked neural response (R1) and the later evoked resonant neuronal activity (ERNA). For these participants, we defined ERNA as an oscillatory signal in response to both anodal and cathodal stimulation at a post-stimulus latency between 3 and 7 ms.
